# The relationship of benevolent sexism and disordered eating in China: the role of appearance comparison on social network sites

**DOI:** 10.3389/fpsyg.2025.1648431

**Published:** 2025-11-13

**Authors:** Ruijie Xu, Changkai Chen

**Affiliations:** 1 Department of Psychology, School of Social and Behavioral Sciences, Nanjing University, Nanjing, China; 2 Center for Mental Health Education and Research, Nanjing University, Nanjing, China

**Keywords:** benevolent sexism, disordered eating, appearance comparison, social network site (SNS), ambivalent sexism

## Abstract

**Introduction:**

This study focuses on disordered eating (DE), a critical mental health issue among university students. Specifically, it examines the relationship between benevolent sexism (BS) and DE, and explores the underlying psychological mechanisms.

**Methods:**

A questionnaire-based survey was conducted to investigate the association between BS and DE, as well as the mediating role of appearance comparison on social networking sites (SNSs) and the moderating role of gender. A sample of 2,000 Chinese college students completed the Ambivalent Sexism Inventory, subscales of the Eating Disorder Inventory, and the Social Network Site Appearance Comparison Scale. Using Hayes’ Process Macro, this study tested both a mediation model (Model 4) and a moderated mediation model (Model 59).

**Results:**

The results showed that BS was positively associated with both appearance comparison on SNSs (*a* = 0.038, *p* < 0.001) and DE (*c* = 0.091, *p* = 0.001). Appearance comparison on SNSs was positively associated with DE (*b* = 1.733, *p* < 0.001) and partially mediated the link between BS and DE (ab = 0.066, 95% CI [0.04, 0.10]). Gender further moderated these associations: BS can directly predict DE among females (c_female_ = 0.207, *p* < 0.001) but not among males (c_male_ = 0.033, *p* = 0.319), and the effect of appearance comparison on SNSs on DE was stronger for females (b_female_ = 1.966, *p* < 0.001) than for males (b_male_ = 1.586, *p* < 0.001). Conditional indirect effects confirmed that the mediation pathway was significant for males (ab_male_ = 0.070, 95% CI [0.04, 0.11]) but not significant for females (ab_female_ = 0.051, 95% CI [−0.01, 0.11]).

**Discussion:**

These results integrate Ambivalent Sexism Theory with Social Comparison Theory, illustrating how BS operates within China’s digital and culrural contexts to heighten the risk of disordered eating. The findings emphasize the necessity of culturally grounded and digitally informed interventions that counter benevolent sexist ideologies and reduce appearance-focused social comparison.

## 1 Introduction

Disordered eating (DE) refers to unhealthy behavior patterns related to diet and food (Otis et al., 1997). In recent years, the prevalence of DE in the Chinese population has increased (Yao et al., 2020; Wu et al., 2022), particularly among young adults (Wu et al., 2022). Approximately 2.5% of Chinese university students had DE in 2010 (Liao et al., 2010), and this figure had risen to 10.4% by 2023 (Wang et al., 2023). Given the severe mental and physical consequences of disordered eating, it is essential to examine not only its epidemiological trends but also the sociocultural mechanisms that underpin its development.

Ambivalent Sexism Theory (AST) distinguishes between hostile sexism (HS), which refers to overtly negative and antagonistic attitudes toward women, and benevolent sexism (BS), which appears subjectively positive but is patronizing in nature (Glick and Fiske, 1996). While originally developed to explain the maintenance of gender inequality, AST may help to understand the sociocultural expectations and prescriptive appearance norms that may increase the salience of appearance for self-evaluation. BS idealizes women as pure, fragile, and in need of protection, contingent upon their conformity to traditional gender roles (Glick and Fiske, 1996). Moreover, BS prescribes how women should look, rewarding adherence to ideals of thinness and beauty (Forbes et al., 2004), while simultaneously reinforcing complementary masculine ideals for men, such as a muscular physique (Swami and Voracek, 2013). This prescriptive pressure to embody gendered appearance norms can create a fertile ground for body dissatisfaction and maladaptive eating behaviors. While BS may appear flattering, it ultimately undermines women’s autonomy by rewarding conformity and penalizing deviation, thereby reinforcing structural gender inequality (Bareket and Fiske, 2023). Critically, despite its subjectively positive tone, BS is associated with detrimental outcomes, including diminished self-competence and increased anxiety (Dardenne et al., 2013; Jost and Hunyady, 2005), paving the way for its link to body image disturbances. Although most research has focused on HS, growing attention has been directed toward the insidious effects of BS, particularly in the domains of body image and eating behavior.

A robust body of evidence demonstrates a consistent association between BS and body image concerns. Experimental studies have shown that exposure to benevolent sexist messages increases women’s willingness to pursue appearance modification to align with prescriptive gender norms (Calogero and Jost, 2011). BS has also been linked to heightened body dissatisfaction (Forbes et al., 2007; Martínez-Marín et al., 2025). Moreover, research has shown that women’s endorsement of BS was more strongly correlated with body dissatisfaction than was hostile sexism (Forbes et al., 2004). Such body dissatisfaction is a well-established precursor of disordered eating behaviors such as restrictive dieting, binge eating, and purging (Mallaram et al., 2023; Jackson et al., 2022). Taken together, these findings indicate that BS helps to perpetuate appearance-based standards of worth. However, whether and how this sociocultural pressure translates into DE remains underexplored.

While most research has centered on women, BS also exerts pressures on men. Men who endorse benevolent sexist attitudes may internalize hegemonic masculine ideals, experiencing pressure to embody a muscular physique in order to fulfill the protector role complementary to women’s “fragility” (Swami and Voracek, 2013). This drive for muscularity has been linked to excessive exercise, restrictive diets, and other disordered eating behaviors among men (Murray et al., 2017). However, relatively little research has examined how BS may predispose men to engage in body image comparisons, particularly within digital environments. Addressing this gap is critical, as understanding gender-specific pathways can provide a fuller picture of how BS influences eating pathology.

A key psychological mechanism connecting BS to DE may be appearance-based social comparison. According to Social Comparison Theory (Festinger, 1954), individuals evaluate themselves by comparing with others. When an attribute becomes central to one’s social identity or self-worth, Social Comparison Theory predicts heightened engagement in upward and lateral comparisons to evaluate one’s standing. In the context of BS, which emphasizes adherence to gendered appearance norms, individuals may be more motivated to engage in appearance comparisons to assess whether they meet these idealized standards (Franzoi, 2001). This process is not limited to offline contexts. With the rapid expansion of social networking sites (SNSs), social comparison processes are increasingly enacted in digital spaces (Verduyn et al., 2020). Visual platforms such as Instagram and TikTok amplify opportunities for upward appearance comparison, as users are constantly exposed to curated and idealized images (Tiggemann and Slater, 2013; Duguay, 2016). A growing body of research confirms that appearance comparison on SNSs is a significant predictor of body dissatisfaction and disordered eating (Fardouly et al., 2015; Holland and Tiggemann, 2016). In China, online media exposure and fitness management app use also play a crucial role in the generation of disordered eating symptoms in young adults, especially in females (Guo et al., 2022).

Although BS has been linked to body dissatisfaction, few studies have directly examined the relationship between BS and DE. The mechanism like the specific role of appearance comparison as a mediator is even more untested. If BS reinforces gendered appearance norms, it follows that individuals who endorse these beliefs may be more vigilant and attentive to social cues regarding appearance, making them more prone to engage in appearance comparisons on SNSs where such cues are abundant. Moreover, prior research has largely focused on Western populations and women, leaving little understanding of how these processes unfold among Chinese young adults navigating the tension between traditional cultural values infused with benevolent sexist attitudes and globalized beauty standards proliferated via social media. Finally, the pathways for men remain underexplored, despite evidence suggesting that BS may drive pressures toward muscularity and related appearance comparisons.

The present study may address these gaps by testing an integrated model in which BS predicts DE through appearance comparison on SNSs among Chinese university students. This study aims to extend theoretical work on benevolent sexism and social comparison into the digital age. By integrating Ambivalent Sexism Theory with Social Comparison Theory within the digital context and applying it to both men and women, this research may contribute to a more comprehensive understanding of how sociocultural gender ideologies intersect with modern media environments to shape DE. It also provides culturally contextualized evidence from China, offering insights for prevention and intervention efforts targeting university students.

## 2 Materials and methods

### 2.1 Participants and procedure

Participants were recruited from a key university in Eastern China. Participants of all genders were eligible to participate. A cluster sampling strategy was employed based on college and grade level. The survey was administered through the university’s mental health management system. Informed consent was obtained at the beginning of the survey, participation was voluntary, and the research team’s contact information was provided at the end of the questionnaire.

A total of 2,172 questionnaires were collected. After excluding 139 incomplete responses (e.g., straight-lining responding, obvious random patterns) and 33 responses with a completion time of less than 3 min, 2,000 valid questionnaires remained, yielding an effective response rate of 93.6%. The average age of participants was 20.94 years (SD = 3.46). The sample included 776 females (38.8%) and 1,224 males (61.2%). Other demographic characteristics were as follows: average BMI (calculated from self-reported height and weight) was 21.87 kg/m^2^ (SD = 4.29); 82.0% identified as heterosexual, 2.3% as homosexual, 9.0% as bisexual; 73.9% were single, 26.1% were in a relationship, and the remaining participants chose “other” or preferred not to say.

### 2.2 Measures

#### 2.2.1 Benevolent sexism

The Ambivalent Sexism Inventory (ASI) developed by Glick and Fiske (1996) was used to measure participants’ hostile and benevolent sexist attitudes toward women. The scale consists of 22 items rated on a 6-point Likert scale (1 = strongly disagree to 6 = strongly agree), with higher scores indicating higher levels of ambivalent sexism. It includes two subscales: Hostile Sexism (HS; 11 items, e.g., “Women are always trying to control men by using their sexuality”) and Benevolent Sexism (BS; 11 items, e.g., “Women should be cherished and protected by men”). The Chinese version of ASI has been widely used in previous studies and demonstrated good reliability and validity. In this study, Cronbach’s α was 0.67 for the BS subscale and 0.90 for the HS subscale. The measure is validated for use with both men and women.

#### 2.2.2 Disordered eating

The Eating Disorder Inventory (EDI-I; Garner and Polivy, 1983) was used to assess participants’ disordered eating (DE), focusing on three core subscales: Bulimia (B), Drive for Thinness (DT), and Body Dissatisfaction (BD). The subscales include a total of 23 items rated on a 6-point scale (1 = never to 6 = always) with higher scores indicating a higher risk of eating disorders. This scale and its subscales have been extensively used and validated in both Chinese and international research. In the current sample, Cronbach’s α for the composite disordered eating score was 0.88.

#### 2.2.3 Appearance comparison on social network sites (SNSs)

The Social Network Site Appearance Comparison Scale (SNSACS), adapted by Wei et al. (2017) from the Facebook Appearance Comparison Scale, was used to measure levels of body image comparison on social networking sites. It contains 3 items (e.g., “When I use social networking sites, I compare my physical appearance with others”) rated on a 5-point Likert scale (1 = never to 5 = always), with higher scores indicating higher levels of appearance comparison on social networks. Cronbach’s α for this scale was 0.90 in the present study. The scale is applicable to both male and female populations.

### 2.3 Data analyses

Data were analyzed using IBM SPSS software version 26.0. First, descriptive statistics (including means, standard deviations, and frequencies) were calculated to summarize the sample characteristics and the distribution of key variables. Subsequently, the internal consistency of the scales was assessed through reliability analyses, such as Cronbach’s α coefficient. Pearson correlation analysis was then employed to explore the relationships among benevolent sexism, hostile sexism, appearance comparisons on SNSs, and disordered eating. In addition, a multivariate analysis of variance (MANOVA) was conducted to examine gender differences across hostile sexism, benevolent sexism, disordered eating, and appearance comparisons on SNSs, followed by independent samples *t*-tests as *post hoc* analyses for each dependent variable.

Hayes’ (2013) Process Macro Model 4 was used to test the mediation model. The independent variable (X) was benevolent sexism, the dependent variable (Y) was disordered eating, the mediator was appearance comparison on SNSs. The controlling variables were gender, age, BMI, self-rated health, sexual orientation, relationship status and hostile sexism. Finally, Hayes’ (2013) Process Macro Model 59 was used to test the moderated mediation model. The independent variable (X) was benevolent sexism, the dependent variable (Y) was disordered eating, the mediator was appearance comparison on SNSs, and the moderator (W) was gender. The controlling variables were age, BMI, self-rated health, sexual orientation, relationship status and hostile sexism. Statistical significance was determined when the 95% bias-corrected confidence interval based on 1,000 bootstrap samples did not include 0.

## 3 Results

### 3.1 Common method bias test

Given that all data in this study were collected via self-report, common method bias was a potential concern. To assess this, Harman’s single-factor test was conducted by performing an unrotated principal component analysis on all items. The analysis extracted nine factors with eigenvalues greater than 1. The first factor accounted for 19.21% of the total variance, which is below the commonly accepted threshold of 30%. These results suggest that common method bias is not a serious threat in the current data.

### 3.2 Descriptive statistics and correlation analysis

Table 1 presents the means, standard deviations, and correlations of the primary variables in this study. On average, male participants reported higher levels of hostile sexism (HS), while exhibiting lower levels of appearance comparisons on social networking sites (SNSs), and disordered eating (DE) compared to female participants.

**TABLE 1:** Descriptive statistics and correlation matrix (*N* = 2000).

Variables	1	2	3	4
1 Benevolent sexism	1			
2 Hostile sexism	0.018	1		
3 Disordered eating	0.107**	−0.048*	1	
4 Appearance comparison on SNSs	0.096**	0.022	0.491**	1
*M*	33.17	31.55	31.76	6.06
SD	7.73	11.03	10.87	2.81

N, sample size; M, mean; SD, standard deviation. *p < 0.05, **p < 0.01.

Partial correlation analyses were conducted while controlling for gender, age, BMI (calculated as weight in kilograms divided by height in meters squared), sexual orientation, and relationship status. Results showed that benevolent sexism (BS) was significantly positively correlated with appearance comparison on SNSs, and DE (ps < 0.05). In contrast, HS was not significantly associated with any of the main outcome variables (ps > 0.05).

### 3.3 Gender differences

To examine gender differences across multiple dependent variables simultaneously, a multivariate analysis of variance (MANOVA) was conducted with HS, BS, DE, and appearance comparison on SNSs as outcomes. The multivariate effect of gender was significant, Wilks’ λ = 0.561, *F*(4, 1995) = 390.275, *p* < 0.001, partial η^2^ = 0.439.

Follow-up independent samples *t*-tests revealed that males scored significantly higher on HS (*M* = 37.09, SD = 9.19) than females (*M* = 22.77, SD = 7.34), *t*(1998) = −38.59, *p* < 0.001. No significant gender difference emerged for BS (M_male_ = 33.06, SD = 8.01; M_female_ = 33.31, SD = 7.28), *t*(1998) = 0.76, *p* = 0.467 > 0.05.

Regarding DE, females reported significantly higher levels (*M* = 34.71, SD = 11.25) than males (*M* = 29.89, SD = 10.19), *t*(1998) = 9.67, *p* < 0.001. Similarly, females engaged more frequently in appearance comparisons on SNSs (*M* = 6.44, SD = 2.86) compared to males (*M* = 5.81, SD = 2.76), *t*(1998) = 4.91, *p* < 0.001.

### 3.4 The mediating role of appearance comparison on SNSs

A simple mediation analysis was conducted using Model 4 of the PROCESS macro (version 4.2) for SPSS to examine whether appearance comparison on SNSs mediated the relationship between BS and DE. Covariates included gender, age, BMI, self-rated physical health, sexual orientation, relationship status, and HS. The overall regression model was significant, R^2^ = 0.29, *F*(9, 1990) = 89.87, *p* < 0.001.

Specifically, BS was positively associated with appearance comparison on SNSs (*a* = 0.038, SE = 0.008, *p* < 0.001) and DE (*c* = 0.091, SE = 0.027, *p* = 0.001). Appearance comparison on SNSs was positively associated with DE (*b* = 1.733, SE = 0.075, *p* < 0.001).

Bootstrapping analysis confirmed the significance of the indirect effect via appearance comparison on SNSs, ab = 0.066, Boot SE = 0.016, 95% CI [0.04, 0.10], indicating a significant mediating effect. The indirect effect accounted for 44.55% of the total effect, *ab/*(*ab* + *c*) = 42.09%.

### 3.5 The moderating role of gender

A moderated mediation analysis was conducted using Model 59 of the PROCESS macro (version 4.2) for SPSS. BS was entered as the independent variable, DE as the dependent variable, and appearance comparison on SNSs as the mediator. Gender was examined as a moderator. Age, BMI, self-rated physical health, sexual orientation, relationship status, and HS were included as covariates. The overall moderated mediation model was significant, R^2^ = 0.30, *F*(11, 1988) = 75.67, *p* < 0.001.

The path from BS to appearance comparison on SNSs was significant (*a* = 0.035, SE = 0.009, *p* < 0.001). The interaction between BS and gender on appearance comparison on SNSs was not significant (*a*’ = 0.009, SE = 0.009, *p* = 0.273), which means this association did not differ by gender.

**FIGURE 1:**
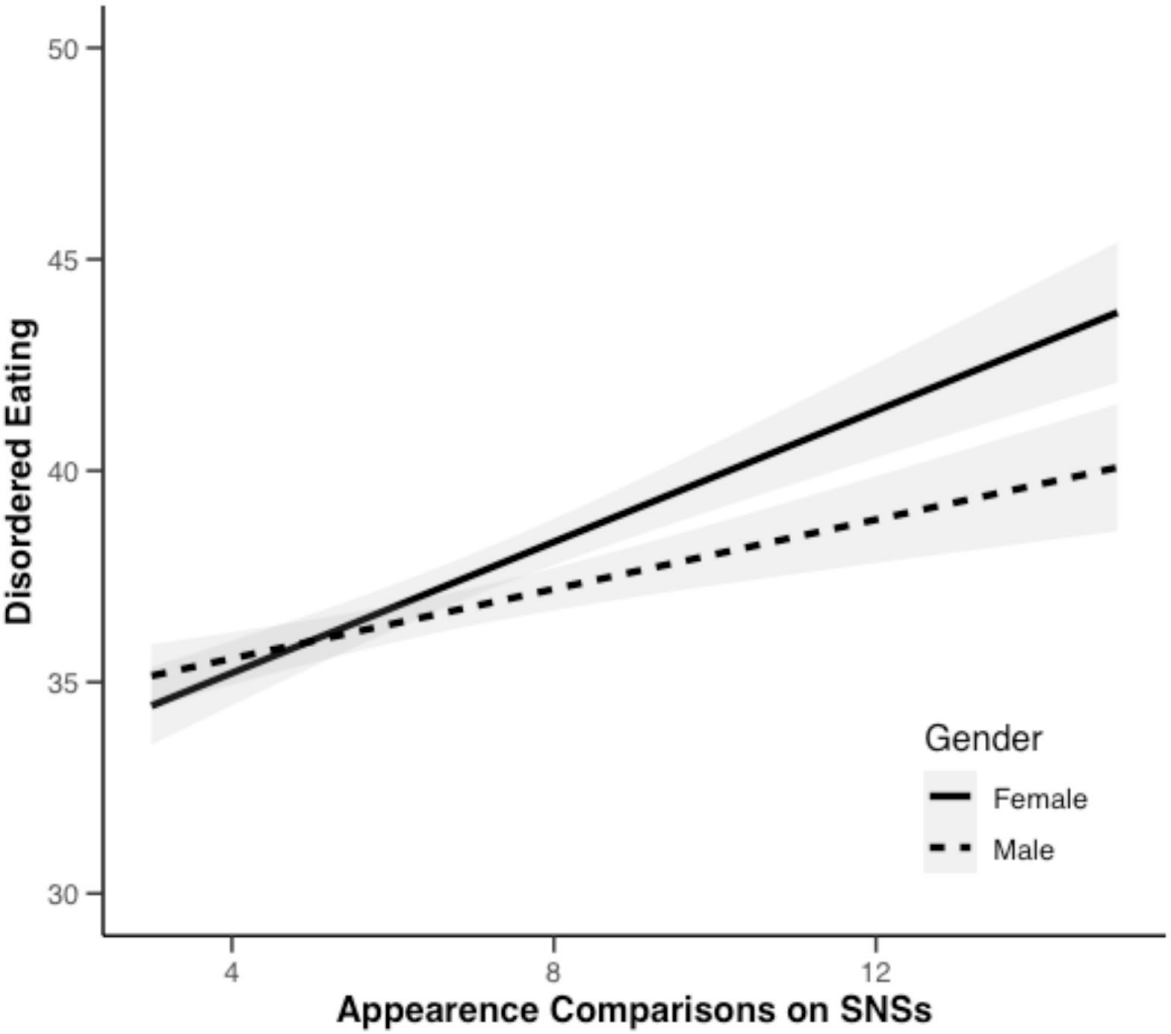
Moderating effect of gender on the relationship between appearance comparison on SNSs and DE.

However, gender moderated the path from appearance comparison on SNSs to DE (see Figure 1), *b*’ = −0.190, SE = 0.075, *p* = 0.012. Appearance comparison on SNSs significantly predicted DE for both females (b_female_ = 1.966, SE = 0.117, *p* < 0.001) and males (b_male_ = 1.586, SE = 0.096, *p* < 0.001), though the effect was stronger among females.

**FIGURE 2:**
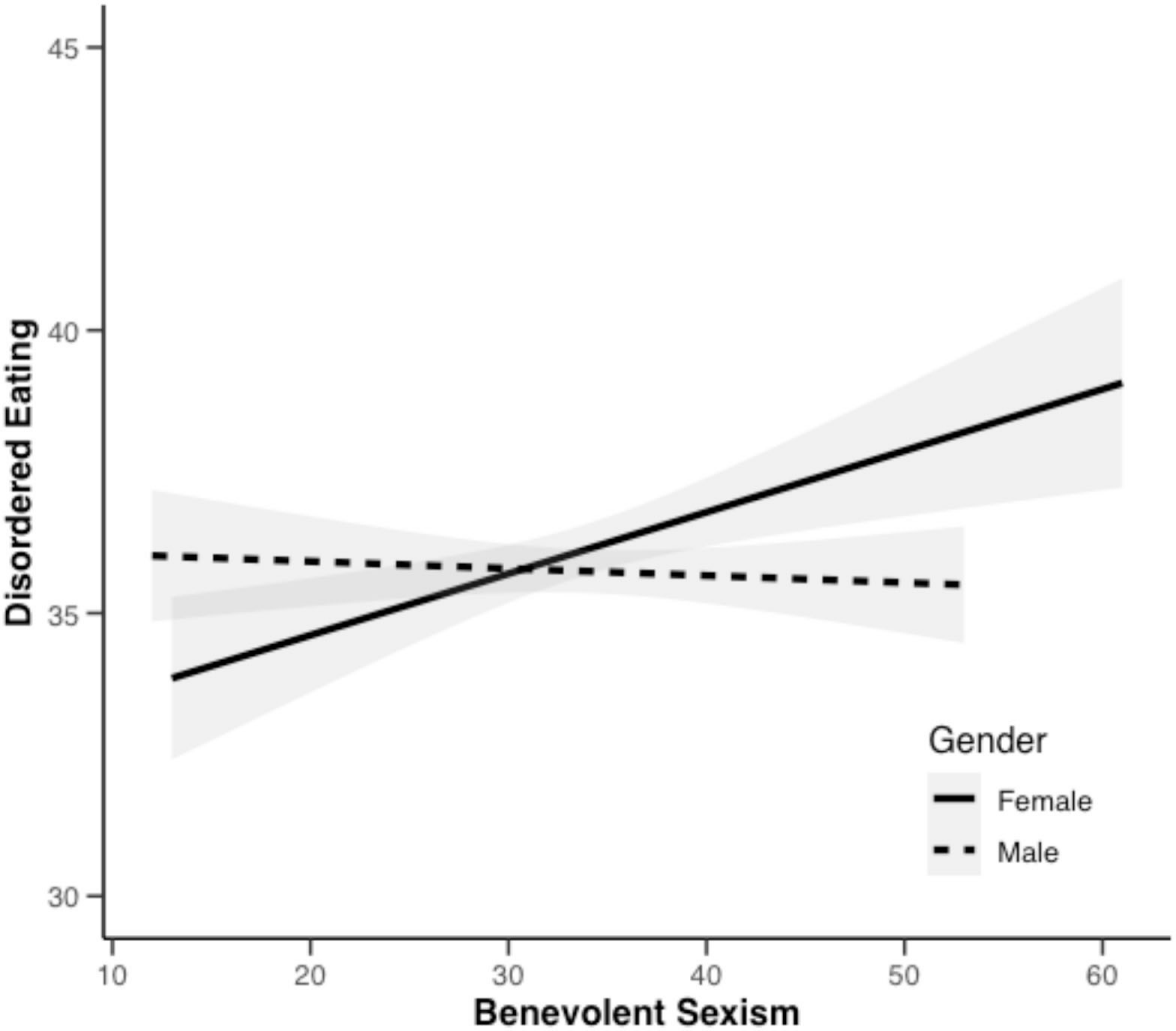
Moderating effect of gender on the relationship between BS and DE.

Gender also moderated the direct path from BS to DE (see Figure 2), *c*’ = −0.087, SE = 0.028, *p* = 0.002. Conditional effect analyses indicated that BS significantly predicted DE among females (c_female_ = 0.207, SE = 0.047, *p* < 0.001), but not among males (c_male_ = 0.033, SE = 0.033, *p* = 0.319 > 0.05).

**FIGURE 3:**
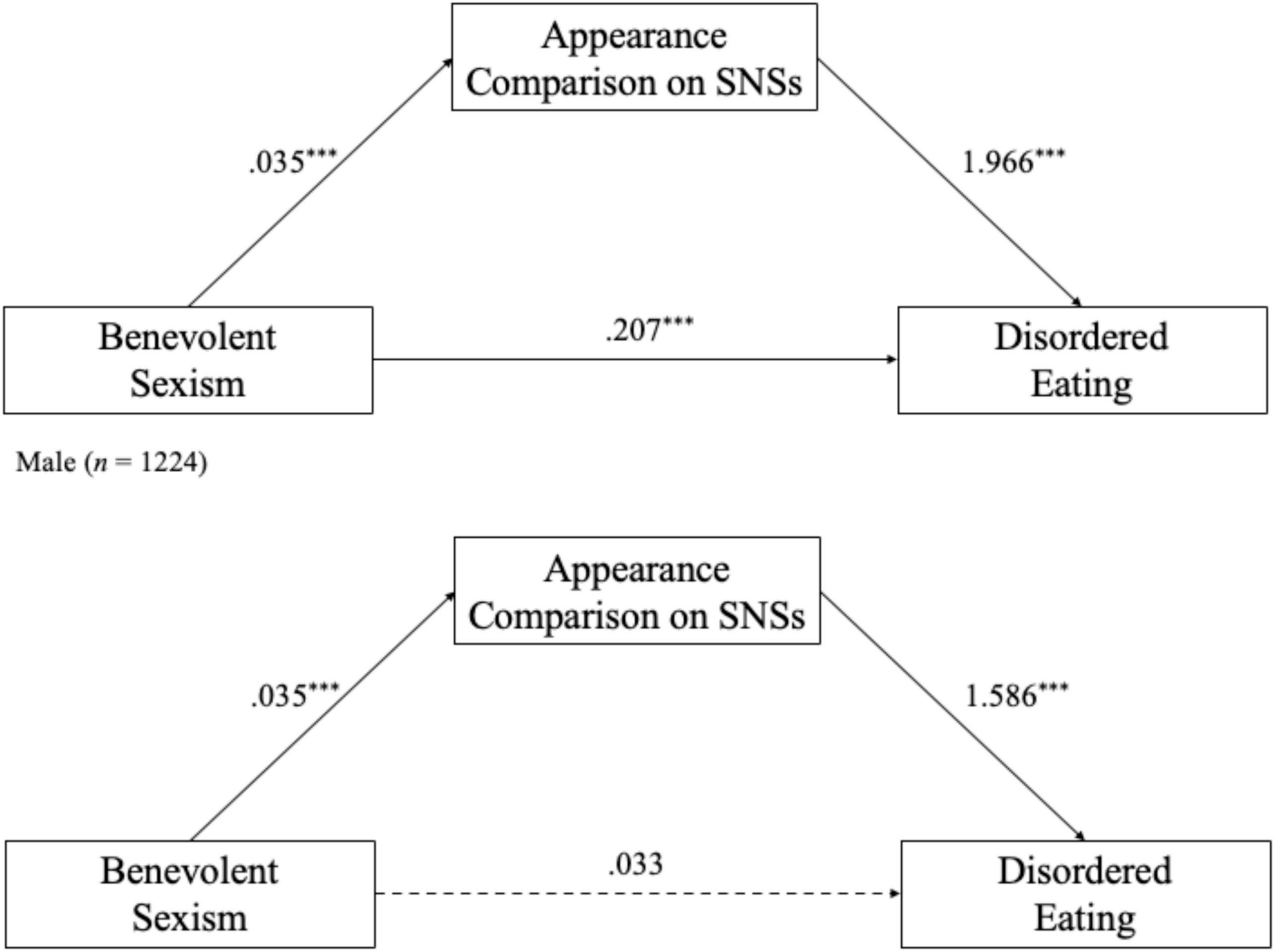
Mediation models for male and female participants. ***p < 0.001.

The conditional indirect effects of BS on DE via appearance comparison on SNSs was significant for males (ab_male_ = 0.070, Boot SE = 0.017, 95% CI [0.04, 0.11]), accounting for 63.45% of the total effect. For females, the indirect effect was not significant (ab_female_ = 0.051, Boot SE = 0.032, 95% CI [–0.01, 0.11]). Figure 3 presents the final moderated mediation models by gender.

## 4 Discussion

The present study investigated the psychological mechanisms linking benevolent sexism (BS) to disordered eating (DE) among Chinese university students, with particular attention to the mediating role of appearance comparison on social networking sites (SNSs) and the moderating role of gender. This work contributes to the growing body of literature examining the subtle yet pervasive influence of BS on body image and eating behaviors, thereby addressing several gaps identified in prior research.

Consistent with the central proposition of Ambivalent Sexism Theory (Glick and Fiske, 1996), our findings demonstrate that BS, despite its seemingly protective rhetoric, is significantly associated with negative psychological outcomes. Importantly, this study responds to the relative paucity of direct evidence connecting BS to DE, underscoring BS as a sociocultural risk factor for problematic eating across genders. This extends prior studies that have primarily linked BS to negative psychological outcomes like anxiety (Barreto and Doyle, 2022) and interpersonal outcomes like unreasonable division of family obligation (Hammond and Overall, 2013) by showing its relevance to individual mental health and maladaptive behaviors.

In contrast, hostile sexism (HS) showed a small but significant negative correlation with disordered eating. Although not a primary focus of this study, this finding is noteworthy and aligns with some prior evidence suggesting that overt antagonism toward women may be less directly linked to the internalization of appearance-related pressures than BS (Swim et al., 2001). The negative correlation may reflect complex dynamics, potentially moderated by gender, and warrants further investigation in future research.

The present study further identifies appearance comparison on SNSs as a key mechanism through which BS exerts its influence on DE. This finding can be effectively understood through the Social Comparison Theory, which posits that individuals evaluate themselves by comparing with others, particularly in domains of high personal relevance (Festinger, 1954), such as physical appearance. Whereas Social Comparison Theory originally emphasized self-evaluation in the absence of objective standards, our results suggest that these processes may be amplified and externalized through digitized cycles of appearance comparison, peer feedback, and algorithm-driven exposure to idealized images (Fardouly et al., 2017; Harriger et al., 2022). By showing that BS operates through appearance comparison on SNSs, our findings bridge Ambivalent Sexism Theory with Social Comparison Theory in a way that highlights the evolving sociocultural pressures of contemporary digital contexts. Recent longitudinal network analysis among Chinese adolescents also confirms that upward physical appearance comparison is a strong predictor of eating disorder symptoms, especially for girls (Zhang et al., 2024), underscoring the relevance of social comparison mechanisms in Chinese digital contexts.

Effect sizes for the association between BS and appearance comparison on SNSs were relatively small. This suggests that BS may represent only one of multiple sociocultural influences on body image concerns. Other forces, such as peer norms, mass media portrayals, and broader cultural ideals, may exert stronger effects (Merino et al., 2024). These results underscore the necessity of adopting a multifactorial approach when examining DE.

Gendered analyses revealed both convergences and divergences in these pathways. For men, appearance comparison on SNSs fully mediated the link between BS and DE. Although benevolent sexism is ostensibly directed toward women, men who endorse these beliefs may also internalize complementary masculine ideals, such as a more muscular physique (Swami and Voracek, 2013). This endorsement may encourage upward comparisons with hypermasculine figures on social media (e.g., fitness influencers), potentially leading to muscle dissatisfaction and DE practices such as rigid dietary control or excessive exercise (Griffiths and Murray, 2025; Murray et al., 2017; Bordo, 1999). These results add to the relatively scarce literature on BS and male body image, showing that the ideology exerts indirect effects on men by shaping their internalization of hegemonic masculinity.

For women, the model indicated partial mediation, with a direct effect of BS on DE persisting. This suggests that beyond appearance comparison on SNSs, BS may exert influence through alternative pathways, such as direct internalization of prescriptive beauty norms or heightened self-objectification in offline settings (Calogero and Jost, 2011). Interestingly, the path from BS to appearance comparison on SNSs was significant for both men and women. This result may reflect that university students’ engagement in appearance comparison on SNSs is so pervasive and influenced by diverse sociocultural pressures that gender does not add unique explanatory variance (Harriger et al., 2022). However, BS may function as a stronger ideological marker of gender-norm conformity, thereby more directly predicting appearance comparison behaviors, which need further confirmation in future research.

A clarification regarding the moderating role of gender is necessary to interpret the results accurately. Our analysis yielded a nuanced pattern. The index of moderated mediation, which is the definitive statistical test for whether the strength of the indirect effect differs across sexes, was significant. This indicates that the difference in the mediation pathway via appearance comparison on SNSs between males and females was statistically reliable. Moreover, gender was found to moderate two specific direct paths within the model. First, the direct path from BS to DE was significant for females but not for males. Second, the path from appearance comparison on SNSs to DE was significantly stronger for females than for males. It suggests that gender influences specific components of the process differently. This pattern underscores the complexity of gender’s role and highlights the value of examining specific pathways in addition to the overall mediated effect.

The study’s Chinese context also provides important cultural insights. Prior research has been predominantly Western, where ambivalent sexism reflects patriarchal structures rooted in individualistic cultures. In contrast, Confucian traditions often frame women’s domestic and familial obligations as moral virtues (Chen et al., 2022), potentially altering the interpretation and impact of BS. For Chinese university students, BS may be more normalized and less likely to be perceived as overtly discriminatory (Chen et al., 2022), yet still fosters conformity to appearance-related norms. Moreover, the high penetration of social media in Chinese student life may amplify the digitized mechanisms of social comparison more strongly than in some Western samples (Bai et al., 2024). By highlighting these contextual dynamics, this study underscores the need for culturally sensitive adaptations of ambivalent sexism measures and culturally grounded interpretations of their effects.

Theoretically, the findings advance understanding in three ways. First, they extend Ambivalent Sexism Theory by demonstrating that benevolent sexism contributes to disordered eating behaviors, thereby broadening its implications beyond interpersonal outcomes. Second, they expand Social Comparison Theory into the digital age by situating appearance comparison within SNS-based cycles of comparison and algorithmic reinforcement (Harriger et al., 2022). Third, they integrate perspectives on gender, culture, and digital media, highlighting the intersectional nature of body image concerns.

Practically, the results suggest several directions for intervention. First, efforts should target both sociocultural ideologies (e.g., challenging benevolent sexist beliefs) and individual-level processes (e.g., reducing appearance comparison behaviors). Second, awareness campaigns should address the covert harms of benevolent sexism, which may otherwise be overlooked due to its superficially positive tone (Chen et al., 2022). Third, social media platforms should assume responsibility for mitigating appearance-related harms by reducing the promotion of idealized body content and fostering exposure to body-diverse, body-positive narratives (Mazzeo et al., 2024).

Several limitations should be noted. First, as the study employed a cross-sectional design, these associations should be interpreted as correlational rather than causal. Future longitudinal or experimental studies are needed to verify whether BS indeed contribute to the onset or maintenance of DE. Second, the sample was drawn from a single university, limiting generalizability. Broader samples across regions and age groups are needed. Third, while we employed a standardized measure of DE (EDI-I), future studies should refine and validate localized instruments to better capture culturally specific eating concerns and gender ideologies. Finally, the ambivalent sexism framework, developed in Western contexts, may not fully capture the nuances of Chinese cultural norms. Future work should incorporate culturally specific items (e.g., marriage pressure, son preference) and examine intersections between individual beliefs, familial obligations, and sociopolitical structures.

## Data Availability

The raw data supporting the conclusions of this article will be made available by the authors, without undue reservation.
